# Considerations for Upright Particle Therapy Patient Positioning and Associated Image Guidance

**DOI:** 10.3389/fonc.2022.930850

**Published:** 2022-07-29

**Authors:** Lennart Volz, Yinxiangzi Sheng, Marco Durante, Christian Graeff

**Affiliations:** ^1^ Biophysics, GSI Helmholtz Center for Heavy Ion Research GmbH, Darmstadt, Germany; ^2^ Department of Medical Physics, Shanghai Proton and Heavy Ion Center, Shanghai, China; ^3^ Institute of Condensed Matter Physics, Technical University of Darmstadt, Darmstadt, Germany; ^4^ Institute of Electrical Engineering and Information Technology, Technical University of Darmstadt, Darmstadt, Germany

**Keywords:** upright CT, upright treatment, particle therapy, image guidance, seated treatment, treatment chair

## Abstract

Particle therapy is a rapidly growing field in cancer therapy. Worldwide, over 100 centers are in operation, and more are currently in construction phase. The interest in particle therapy is founded in the superior target dose conformity and healthy tissue sparing achievable through the particles’ inverse depth dose profile. This physical advantage is, however, opposed by increased complexity and cost of particle therapy facilities. Particle therapy, especially with heavier ions, requires large and costly equipment to accelerate the particles to the desired treatment energy and steer the beam to the patient. A significant portion of the cost for a treatment facility is attributed to the gantry, used to enable different beam angles around the patient for optimal healthy tissue sparing. Instead of a gantry, a rotating chair positioning system paired with a fixed horizontal beam line presents a suitable cost-efficient alternative. Chair systems have been used already at the advent of particle therapy, but were soon dismissed due to increased setup uncertainty associated with the upright position stemming from the lack of dedicated image guidance systems. Recently, treatment chairs gained renewed interest due to the improvement in beam delivery, commercial availability of vertical patient CT imaging and improved image guidance systems to mitigate the problem of anatomical motion in seated treatments. In this review, economical and clinical reasons for an upright patient positioning system are discussed. Existing designs targeted for particle therapy are reviewed, and conclusions are drawn on the design and construction of chair systems and associated image guidance. Finally, the different aspects from literature are channeled into recommendations for potential upright treatment layouts, both for retrofitting and new facilities.

## Introduction

Particle therapy, available in the form of proton or heavier ion therapy, has received an ever-increasing interest over recent decades, with currently over 100 facilities in operation (1), and several more planned or under construction. Primarily, this interest results from the superior depth dose profile of charged particles over that of photons used in conventional radiotherapy: particles have a finite range in the patient and deposit the maximum dose at their stopping point - the Bragg peak. The Bragg peak enables delivery of a high dose to the target with minimum dose to surrounding healthy tissue. Sufficient target coverage can already be achieved with few treatment fields, therefore the low-dose bath typically delivered to a large volume for photon therapy. In case of heavier ions, the Bragg peak is also the point of highest relative biological effectiveness (RBE), further increasing tumor dose relative to that in healthy tissue (2).

This physical advantage of particle therapy over photon therapy, however, is opposed by the increased capital investment required for a particle therapy facility. This is attributed to the larger and more complex accelerator equipment needed to produce medical ion beams. Especially for heavy ions, a large part of the overall investment is needed for the gantry, used to enable treatment from different angles around the patient (3). It is therefore not surprising that several groups have been, or are currently, investigating options for gantry-less particle therapy, including treatment delivery with the patient in an upright or seated position. In fact, for the pioneering studies on ion beam therapy, conducted at the Lawrence Berkeley National Laboratory (LBNL), patients were fixed in an upright position using a rotating chair setup and a vertical CT was installed (4). Gantry-less carbon ion therapy with a custom chair and even associated vertical CT imaging was reported by Kamada et al. (5). A chair system for head and neck patients was also designed at GSI Helmholtz Center for Heavy Ion Research GmbH during its carbon ion therapy pilot project (6). Yet, despite the increased cost and size, gantries are regarded as the best option for enabling flexible beam angles, due to their universal applicability for different treatment sites. The declined interest in chair systems can be attributed to the limited availability of vertical CT systems, and the resulting limited knowledge on anatomical deformations in an upright position.

Indeed, dedicated image guidance is a central aspect when it comes to installing a system to treat patients in the upright position. With the advent of modern image guidance systems (7), like in-room Cone-Beam CT (CBCT), optical surface guidance, as well as dual-energy CT and particle imaging, particle therapy has recently enjoyed a boost in achievable treatment accuracy. Now, with commercial options for vertical CT systems (8, 9), and the drive for reducing the upfront investment for opening new particle therapy centers (3, 10), upright treatment positioning systems have gained renewed interest (11, 12). Moreover, advanced techniques for intra-treatment verification and adaptation (13–15) are currently reaching clinical maturity. These techniques could, not only overcome the previous issues associated with anatomical motion for upright treatment positions, but together with upright treatment postures, further open possibilities for advanced beam delivery schemes, e.g., particle arc therapy (16) with continuous patient rotation.

In this review, we aim to outline key requirements for a flexible upright particle therapy patient positioning system with associated imaging. To this end, we will summarize the clinical rationale for upright treatments and go over existing chair systems that have been constructed in the past or are currently available, focusing on those targeted for particle therapy. An overview over the requirements for an upright positioning system for photon therapy can be found in the recent review by Hegarty et al. (17). We will discuss engineering considerations for upright positioning systems and options for image guidance for an upright treatment position. Finally, we will channel the available literature into recommendations for future upright particle therapy patient positioning systems and associated image guidance.

## Possible Benefit of an Upright Positioning System

### Economical Arguments

The key rationale for upright treatment positioning is, that it is cheaper to move the patient and not the beam. Gantries for proton, and especially for heavier ion therapy, are large, costly pieces of machinery. While it can cost less than half a million Euro to build a head&neck targeted chair positioning system, building a gantry is an investment of several million Euro for protons and even more for heavier ions. There is also operational cost: precise rotation of the up to several hundred ton gantry is by no means a simple task, and requires sophisticated installation and maintenance. For example, a shift of the isocenter position of up to ~1.2 mm during rotation of a proton gantry was reported by Moyers et al. (18). This shift can be effectively compensated by modern scanning delivery systems, but the shift results in additional workload for beam commissioning under different gantry angles. In addition, the large size of a proton, let alone a heavy ion gantry necessitates building a large shielded bunker (up to 25 m in length and three stories height in case of the carbon ion gantry at the Heidelberg Ion-Beam Therapy Center (19)), further adding to the facility cost.

While new designs for compact gantry systems are being explored, e.g., the static toroidal gantry proposed by Bottura et al. (20), moving the patient instead of the gantry presents a possibly simpler and thus attractive option. Especially for existing centers, retrofitting an upright positioning system is conceivably less challenging compared to retrofitting a gantry. Further arguments on cost-reduction could be the potential of faster setup time with an upright positioning system due to easier patient access (21). An upright positioning system may also enable easier inclusion of advanced image guidance techniques, like prompt gamma or particle imaging (13) to the setup, due to more free space around the isocenter.

At the same time, a gantry-less treatment room equipped with a flexible patient positioning system could provide similar versatility compared to a gantry, as demonstrated in the study by Yan et al. (22). An upright positioning system would also naturally provide an efficient way for advanced beam delivery options, like particle arc therapy (16, 23), by rotating the patient during treatment.

Overall, a chair system could be the solution for more widespread access to particle therapy, reducing cost and size of particle therapy centers. An upright proton therapy system has been speculated to possibly fit into a single shielded bunker (3, 24) and could foster greater availability also in low income countries. For heavy ion therapy, where currently only two gantry systems are in operation worldwide, upright patient positioning could be the key to promote this technology from few centers with fixed horizontal beam lines to similar availability levels as current proton therapy.

### Clinical Arguments

Aside from the economical arguments, there have been a small, but growing number of studies indicating a possible therapeutic benefit of upright treatment as well. Upright positioning could provide greater comfort for head&neck patients suffering from increased saliva production (25), and patients suffering orthopnea, dyspnea or dysphagia in supine position (26).

Dellamonica et al. (27) report increased lung volume and oxygenation for upright compared to supine position. Using an upright MRI scanner, Yang et al. (28) also demonstrated increased lung volume in upright positioning compared to supine, as well as showing a reduced motion amplitude from respiration. The greater lung volume implies greater distance between target and organs at risk, as well as lower mean lung dose, which has been used to argue for better healthy tissue sparing possibility (25). For particle therapy, the important quantity is the water equivalent thickness, which would not change relevantly with greater air volume in the lungs. In contrary, the reduced lung density could reduce the dose conformity at the distal target edge. This could be offset by both a reduced breathing amplitude resulting in smaller lateral margins, as well as the aforementioned distances to organs at risk. The net effect is probably patient-specific and needs to be investigated in treatment planning studies.

Considering that patients spend most of their days in upright or seated posture prior to treatment, upright treatment may also provide certain benefits regarding organ drift. For example, slow liver drift motion occurring over tens of minutes after supine positioning of patients was reported by von Siebenthal et al. (29), and attributed to the change in the direction of gravity compared to the patients ‘normal’ upright posture. Similar reasoning could be made also for other organs in the abdomen.

In a recent study based on upright MRI, Mackie et al. (21) have reported benefits for prostate therapy in upright position, with further details available in (30). Gravitational push of organs like the bladder into the pelvic bone reduced uncertainties in prostate position compared to supine. Since gas moves upwards the bowel for upright posture, the risk of unexpected bowel gas movement during prostate therapy possibly might be reduced.

Most recently, Sun et al. (31) presented results from clinical implementation of a chair positioning system targeted for head&neck cancers at the Shanghai Proton and Heavy Ion Center (SPHIC). Of 320 patients treated for head&neck, 15 showed a clear dosimetric benefit compared to a treatment with couch and fixed horizontal or 45° inclined beam line. Chair systems also were shown in the past to be well accepted by the patients. For example, McCarroll et al. (26) reported overall good patient comfort for their prototype chair design, but noted that upright positioning might not be possible for all patients, e.g., due to certain medical conditions.

Yan et al. (24) in a treatment planning study have demonstrated the robustness and high quality of upright plans for head&neck cancer patients for pencil-beam scanning proton therapy at a fixed beam line. While they used only a subset of the beam angles available for gantry treatments for upright planning, the authors note that the use of more beam angles could further benefit organ-at risk sparing.

Chair positioning systems are already well established standard for particle therapy treatments of cancers in the eye (32). If the beam line is capable of handling also the special requirements for eye cancer treatment (33), a head&neck upright positioning system may also be useful for eye treatments. Experience on head fixation devices for eye treatments, like chin bars with mouth pieces for the patient to bite into for immobilization, may be transferable also to treatments of brain tumors, although to a limited extent.

## Brief Overview Over Existing Solutions

**Figure 1:**
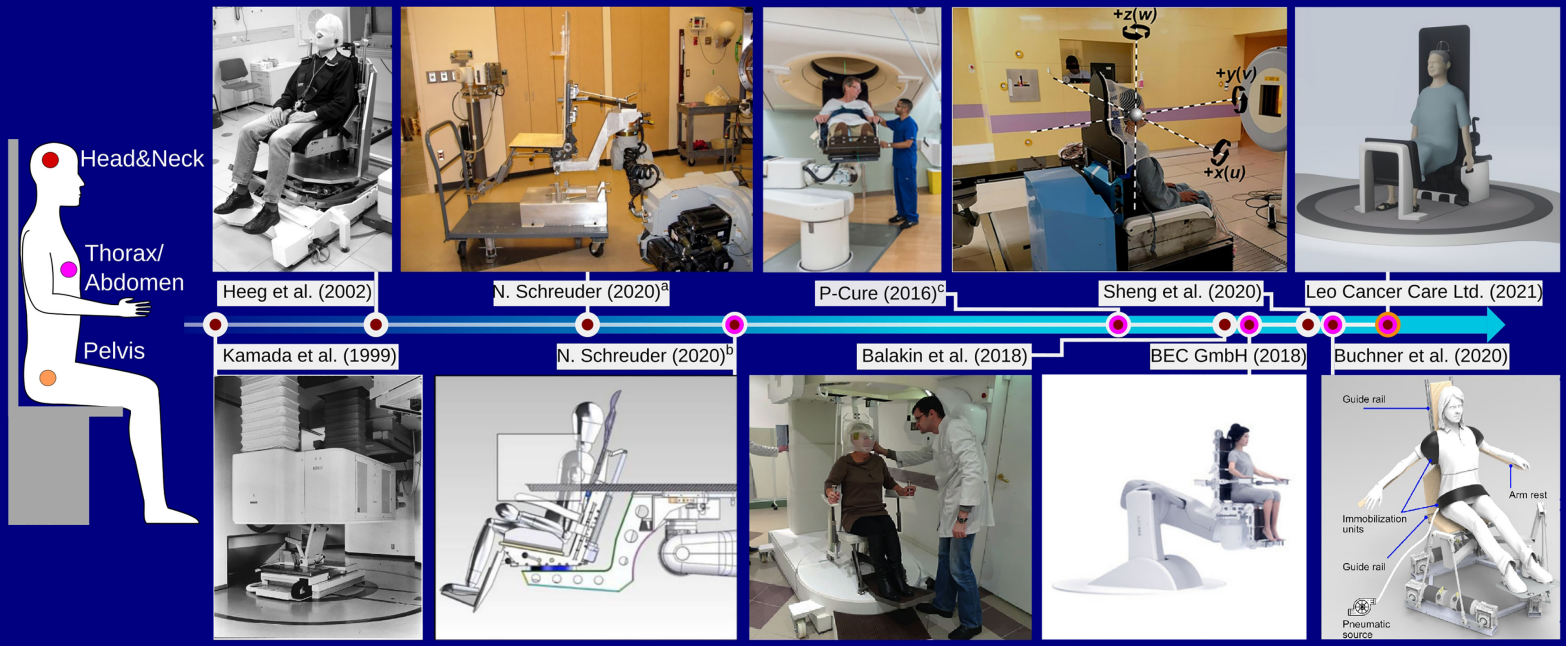
Overview over different chair designs targeted for particle therapy found in literature, focusing on those that were constructed as prototype. The color of the time line connectors indicates the chair’s intended use with respect to different treatment sites, as shown on the left. ^a^The chair was installed at the Indiana University Health Proton Therapy Center prior to 2006, as described by Schreuder (30). ^b^The device was installed at the Oklahoma Proton Center as described in (30). The figure shows the couch overlayed on the chair. ^c^The chair system is installed at Northwestern Medicine Chicago Proton Center and was designed by P-Cure^1^. Image reprinted with kind permission by Dr. M. Pankuch (Northwestern Medicine Chicago Proton Center). Images ^a^,^b^, and the Leo Cancer Care Ltd (2021). were reprinted with kind permission by Dr. N. Schreuder (Leo Cancer Care Ltd.). Kamada et al. (1999): Reprinted from Kamada et al. (5) with permission from Elsevier. Buchner et al. (2020): ^©^2020 IEEE. Reprinted, with permission, from Buchner et al. (35).

In this section, existing solutions for upright positioning devices targeted specifically for particle therapy will be briefly reviewed. **Figure 1** shows examples of upright positioning prototypes constructed for or used at particle therapy centers. A historical overview of upright patient positioning systems for radiotherapy in general can be found in the recent review by Rahim et al. (34).

For the pioneering studies at LBNL investigating particle therapy using various ion species, patients could be positioned in both recumbent and in upright, standing or sitting, treatment position (36, 37). Patient positioning was achieved using dedicated upright accessories added to the ‘ISAH’ (38) 5-degree-of-freedom patient positioning system (36). A vertical CT was used for position verification in the upright position (4)Kamada et al. (5) have presented a chair positioning system for head&neck cancer patients used at the Heavy Ion Medical Accelerator Center (HIMAC) in Chiba, Japan. The device was mounted on a rail system to move it from treatment to parking position and a vertical CT scanner was specifically installed for imaging in the seated position. The CT scanner, mounted to the ceiling of the treatment bunker, was lowered over the patient to acquire images.

Heeg et al. (6) report on the design of a patient chair to be used at the GSI Helmholtz Center for Heavy Ion Research GmbH, with further refinements detailed in (39). The chair enabled a tilt of up to 19°, a 360° rotation around the vertical axis and featured a height adjustable patient mask holder mounted to a steel frame with counterweights for safe handling. Similar to the chair system by Kamada et al. (5), the chair by Heeg et al. (6) was mounted on a rail system to make way for the couch when not in use. The chair system is still operational and located in the now dedicated research room (40) at GSI, but it was never used clinically.

Schreuder (30) discusses the design and construction of a chair system at the Indiana University Health Proton Therapy Center (Bloomington, Indiana, USA), reportedly used for patient treatment prior to the installation of a proton gantry. The device was attached to the couch robot, and could be mounted on a transport cart. Of note is the use of a wooden back plate which was individually manufactured for each patient for accurate positioning. In some cases, patient specific cutouts were made in the back plate to reduce material in the beam path.

Schreuder (30) also reports on the further development of the treatment chair continued at the Oklahoma Proton Center (Oklahoma City, Oklahoma, USA). The follow up device considered a 20° tilted backrest made from carbon fibre. Again, it was equipped with the same coupling structure as also used by the treatment couch, enabling efficient switching between couch and chair. Further design improvement of the chair described in (30) were adopted by P-Cure (P-Cure Ltd., Shilat Industrial Zone, Israel) for a commercial chair and vertical imaging system. The system was installed 2016 at Nothwestern Medicine Chicago Proton Center (NWMCPC, Chicago, Illinois, USA), where it is currently in clinical use. The P-Cure system features a carbon fibre chair with up to 20° tiltable back plate mounted to a robotic arm, capable of moving the chair between isocenter and an in-room vertical x-ray CT scanner (41), also installed at NWMCPC. The chair is targeted for not only head&neck, but also thoracic cancer patients, enabling imaging and treatment down to the diaphragm.

Balakin et al. (42) presented an upright patient positioning system for the Prometheus proton therapy complex at P.N. Lebedev Physical Institute of the Russian Academy of Sciences, Physical Technical Center (Protvino,Russia) the idea of which was proposed in (43). Their design considers an armchair targeted for head&neck cancer patients, that is height adjustable by 500 mm to accommodate patients of different size and enables a full 360° rotation around the vertical axis. Balakin *et al.* report initial experience on patient immobilization.

Recently, Zhang et al. (44) presented design considerations for a patient chair for particle therapy based on a Steward hexapod platform to provide the six-degree-of-freedom (6DOF) motion required for positioning and position correction following image guidance. For improved stability of the device, they propose an additional push rod attached to the center of the treatment chair guiding the motion of the hexapod. In addition, they describe a prototype design for a head&neck immobilization device in upright position with chin support for the patient to rest their head on.

Zhang et al. (45) present extensive design considerations for a patient chair. In an effort to retrofit a patient chair to an existing fixed horizontal beam line with limited available space, they based their design also on a hexapod platform. In their work, detailed stress simulations and experimental validations were performed to ensure highest safety of the components and accuracy of the treatment chair motion under patient load. Clinical implementation of the device with dedicated quality assurance protocol is reported in (46) and first experience with patient treatments is presented in (31).

Buchner et al. (35) report on the design of a novel treatment chair and soft robot immobilization device. Their design is based on a commercially available hexapod platform intended for being used as flight simulator. They developed soft robot immobilization devices to achieve highest positioning accuracy individually adaptable to every patient.

The company BEC GmbH (Pfullingen, Germany), which is offering high-precision robotics solutions for particle therapy, also has a design for a commercial patient chair in their ‘exacure’ radiotherapy portfolio (47), first presented in (48). The chair takes a similar route as the first chair design by Schreuder (30): for allowing high flexibility to accommodate patients and minimize uncertainties in the beam path, the chair is entirely based on small rectangular carbon fiber plates which can be individually removed/arranged.

Finally, another commercial option for an upright positioning system is offered by Leo Cancer Care (Smallfield, Horley, Surrey, UK). Their ambitious design, named EVE™, aims towards enabling upright treatments for head&neck, thoracic/abdominal as well as pelvic treatment sites. The systems’ flexible design will enable different patient postures from sitting to half-standing or standing position. Initial positioning experience with the device was reported in (21).

## Mechanical Design: Requirements and Pitfalls

### General Requirements

The ideal upright patient positioning and image guidance system should enable at least the same treatment flexibility as achievable with a gantry, without compromising on efficiency and accuracy compared to the current standard. An upright positioning system should therefore be:

1. Modular, in order to enable treatments of most patient sites

2. Flexible, in order to provide the optimal positioning and immobilization for all patients

3. Providing sub-millimeter/sub-degree positioning accuracy that remains stable over the treatment duration

4. Capable of quickly correcting the patient position according to image guidance

5. Simple and efficient to use

The first point is perhaps the most challenging, as there will likely always be a percentage of patients for whom the upright treatment position may not be a suitable option. In order to facilitate efficient patient throughput, the patient positioning device should be able to cope with different treatment sites without requiring considerable extra setup changing time.

**Figure 2:**
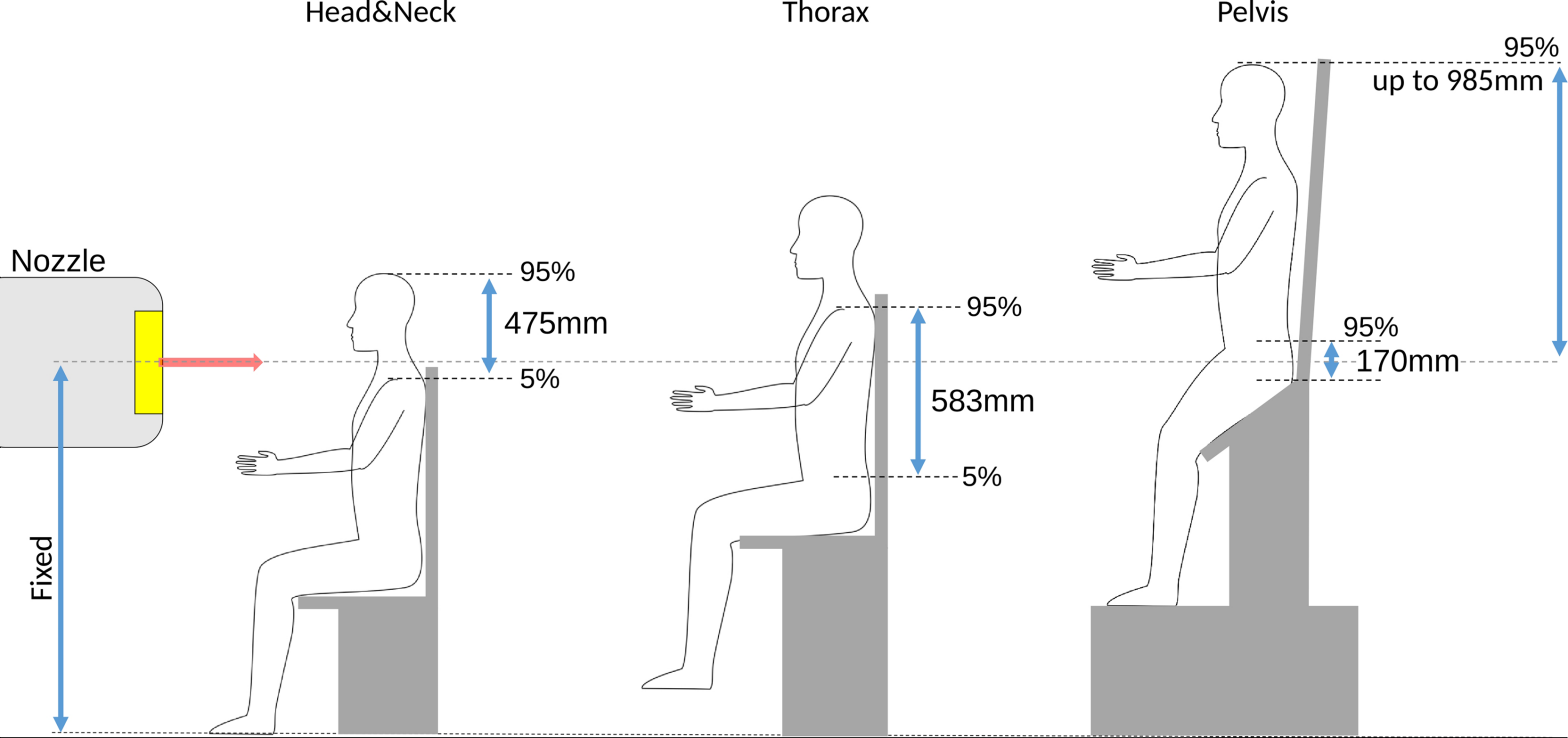
Overview over the required vertical ranges (not to scale) of adjustment for different treatment sites for European adults, computed as the difference between 5 and 95 percentiles from anthropomorphic data in (49). An important constraint for an upright positioning device targeted for a fixed beam line is the fixed height of the isocenter. For pelvis treatments, the patient head can reach up to ~1m above the isocenter.

The need for the positioning and image guidance system to allow flexibility for treating different patient sites, combined with the fixed beam line height poses the key design constraint for an upright positioning system with associated imaging (6, 45). The vertical and lateral translational motion required to adapt the patient positioning system and immobilization devices to the varying height and size of all patients needs to considered. Anthropometric data of European adults for machinery and workplace designs can be found for example in (49). The implications on chair design are indicated in **Figure 2** and summarized in **Table 1**. A straight upright posture of the patient’s back was assumed for calculating required vertical ranges, i.e., rectangular bending angles between tibia/femur and femur/torso. Note that a half standing posture, shown in **Figure 2** as proposed by Leo Cancer Care Ltd (8, 50), would not change the required vertical range of motion of the positioning system to accommodate different treatment sites. It, however, would reduce the weight stress on the chair seat. Lateral ranges assume the rotational center of the patient positioning system to coincide with the lateral geometrical center of the patient. The need to compensate additional eccentricity otherwise adds to the required lateral range of motion.

**Table 1:** Requirements placed on a chair system for different treatment sites.

Treatment site	Head&Neck	Thorax/Abdomen	Pelvis	Total
Height from chair seat (Hi/Lo) [mm]	985/510	695/112	170/0	–
Popliteal height (Hi/Lo) [mm]	–	–	–	495/380
Vertical range [mm]	~475	~583	~170	~985
Lateral range [mm]	±103	±270	±220	±270

Ranges are calculated based on anthropometric data for European adults in (49), reporting the largest value in the range of European adults (i.e., between 5 and/or 95 percentiles). A straight upright sitting posture with rectangular tibia/femur and femur/torso angles was assumed. The range required for pelvis treatments was approximated from the thigh clearance.

For treatment sites below the thorax, the patient needs to be lifted considerably. For treatment of the prostate, the patient’s head may reach up to 985 mm above the isocenter. Enough space above the isocenter is thus mandatory, especially if a vertical CT is to be mounted to the ceiling, which for existing centers may not necessarily be available.

For greatest flexibility, the positioning system should also provide a 360° motion around the vertical axis to enable all treatment directions. Existing prototypes, in addition, consider up to ± 20° tilt around the lateral axis (6, 8, 9, 44, 45). Sufficient experience for the optimal tilt around the lateral axis for different treatment sites is not yet reported in literature, such that the current generation of chair designs may provide more/less flexibility than needed.

In terms of mechanical stability, according to the IEC 60601-1 norm (51), any patient positioning system must be able to safely move a mass of 135 kg plus the weight of accessories. Assuming the weight of the chair and patient immobilization devices (mask, head rest, and connections with chair) to be around 50 kg (45), this necessitates the support structure to comfortably lift 185 kg. In addition, any equipment intended for the patient to step on needs to support at least 270 kg for a minute. These regulations put strong constraints on the engineering choices for the positioning system.

### Considerations on Posture

**Figure 3:**
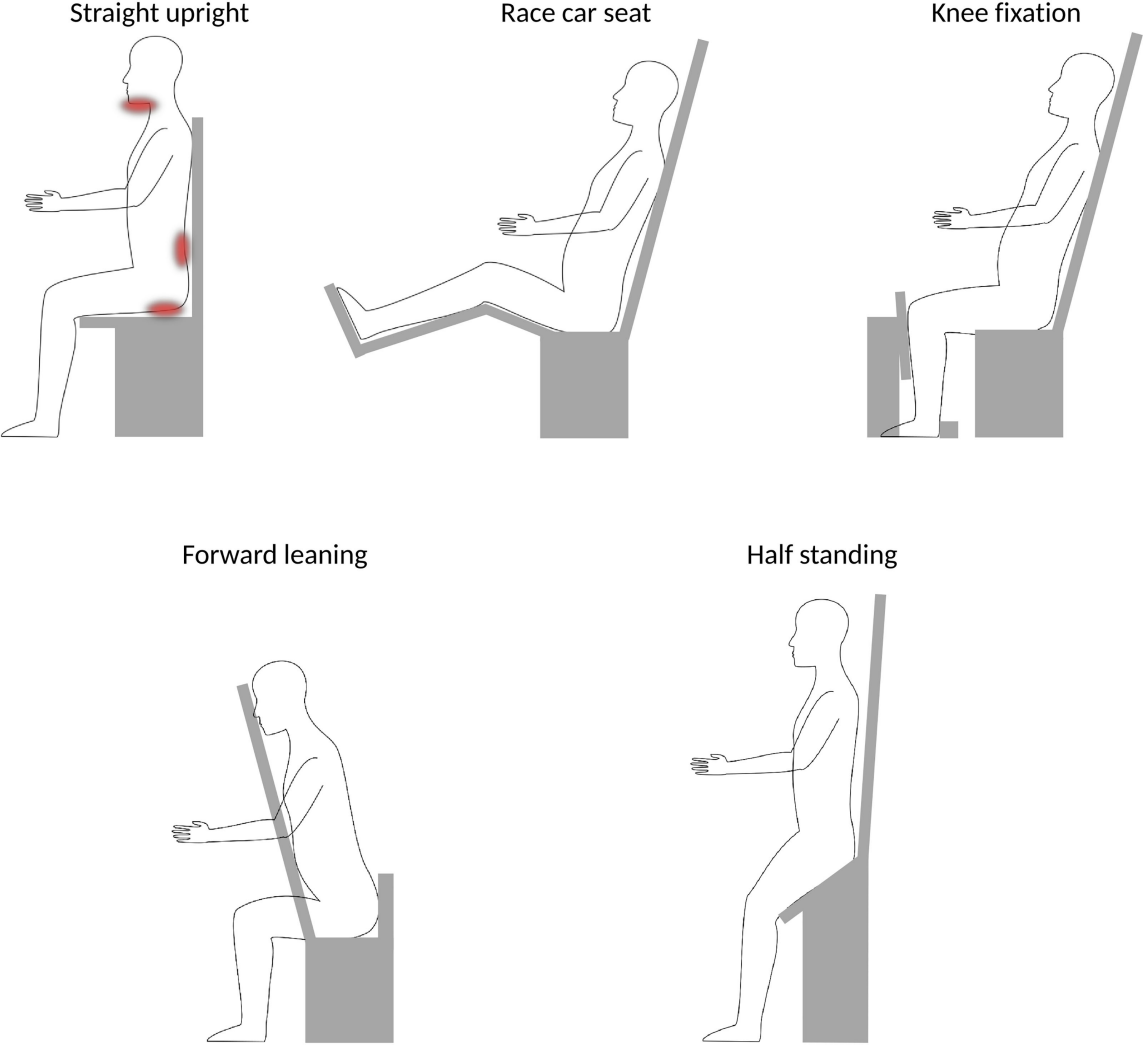
Schematic overview over different postures. A straight upright posture [e.g (42)., and (31)] has been noted to induce stress on chin (for head&neck patients) and may be uncomfortable for long treatment duration, as indicated by the red areas. More ideal would be ~20° reclined position, where the forward push on the pelvis could be stabilized by a race-car seat posture [e.g (9)], or by adding a knee fixation [e.g (8)] or a belt strap [e.g (35)]. Alternative postures could be forward leaning for head&neck or possibly spine irradiations [e.g (26)] or half-standing, enabling to image and treat sites below the thorax [e.g (8)].

Patient posture is important when considering upright treatments, as it can have a significant effect on the patient comfort as well as intra-fraction movement, and has direct implications for the image guidance setup. Different published postures are shown in **Figure 3**.

Sun et al. (31) report patient discomfort for a head&neck chair system, where the patients are positioned in a straight upright position (i.e., a 90° angle between seat and backrest). Patients reportedly tended to sag their heads within the thermoplastic immobilization masks. For long treatments, this may result in increased intra-treatment displacement and patient discomfort. Moreover, especially for long treatments, the comfort for the patients back and bottom becomes important, where e.g. vacuum cushions or simple office chair supply cushions may provide sufficient support. More ideal, however, would potentially be a posture where the back rest is tilted 15-20° backwards pushing the patients into the backrest (21, 31). In fact, most chair solutions for particle therapy consider such a posture (5, 6, 35, 41, 50). Similar findings have been reported by McCarroll et al. (26) who considered a ‘reverse’ chair setup, where the patients are forward leaning against a chest support plate. Additional support for chin and forehead was included in their prototype following initial patient experience, to provide more patient comfort. The forward leaning posture may also be interesting for prone irradiation, for example, in case of treatment of the whole central nervous system.

To provide stability in a leaned-back position, a posture comparable to that in a race car seat (i.e., femur slightly inclined upwards, and bent knees), may be chosen, as done, e.g., by P-Cure Ltd (9, 41). This has the additional benefit that it provides more room for a robot arm underneath the chair, which otherwise could collide with the patient legs (45). Alternatively, a vertical rest for the patients knees to push against, as envisioned by Leo Cancer Care Ltd (8)., may be used to prevent sagging.

For any body site below the thorax, a half standing posture, as proposed by Leo Cancer Care Ltd (8)., is the only viable option. They envision a bending angle between 135° and 165° between femur and torso (50). For seated positions, the patient legs would prohibit a vertical CT scan in the lower body regions.

### 6-Degree-of-Freedom Positioning System

The need to adapt to different patients, treatment sites and beam angles, alongside the need to adapt residual alignment following the image guidance system, necessitates the use of a highly accurate 6DOF support structure. To provide the necessary motion, sequential or linear robotic systems are suitable candidates.

As sequential support structure, a robotic arm as used for patient couches (52) could be used, as for example chosen by P-Cure Ltd (9). The robot arm offers a great flexibility, also in regards of moving the patient from the isocenter to an in-room vertical CT or moving the chair out of the way of other equipment. However, depending on the patient’s posture and the robot position, the patients legs may interfere with the robot (45), limiting the achievable range of motion. Due to its bulky size, retrofitting an additional robot system for an upright positioning system into an existing treatment room may be infeasible alongside the other equipment. Utilizing an available couch robot to also mount the upright positioning system, with a shared coupling mechanism (30), would be the more practical solution.

In contrast, hexapod platforms present a compact solution (35, 44, 45). Hexapods are well suited to support the patient and can achieve high positioning accuracy (45). However, hexapods have a limited range of motion, allowing only restricted position correction when in extreme positions, e.g., when fully vertically extended, and provide a limited range of rotation (44). Zhang et al. (45) circumvented these problems, by adding a translational platform on top of the hexapod and a 360° platform underneath it, effectively increasing the work space to that desired for patient positioning in head&neck cases. In order to provide flexibility for moving the chair to/from the isocenter, a rail system underneath the hexapod may be added as reported in (46).

Alternative designs to robot positioning systems have also been reported in literature. For example, the chair by Heeg et al. (6) features separate mechanisms for rotation, tilt and vertical adjustment in a steel frame. This design was chosen for stability, but is also relatively bulky. The EVE™ design by Leo Cancer Care Ltd (8) considers a height adjustable seat and tiltable back plate to adjust the patient’s back angle. Immobilization devices, like masks and arm rests, will be attached to the back plate. Pitch and lateral position adjustment are achieved by tilting or moving the platform the chair is mounted on. The upright positioning system is placed on a large circular floor platform which provides full 360° rotation and can be lifted to accommodate treatments in lower body regions (abdomen and pelvis).

### Immobilization Devices and Setup Accuracy

Immobilization is crucial especially for particle therapy to conserve beam ranges estimated from planning imaging. For an upright position, in contrast to supine, immobilization devices may need to support part of the patient’s weight. Heeg et al. (39) for their head&neck targeted chair design reported that “the realisation of a secure and precise fixation unit required some rather unconventional details in the construction”. They achieved better than 0.5 mm accuracy by using a steel frame with vertically adjustable mask holder. Steel frames can however, also cause artefacts in x-ray CT.

Zhang et al. (44) report on the development of a head fixation unit with adjustable screws and chin support, but indicated only limited accuracy. Sheng et al. (46) used thermoplastic masks mounted to a carbon fiber back plate for their head&neck targeted chair, which in a later study achieved high patient position accuracy (31). Balakin et al. (42) studied patient movement within thermoplastic masks for head&neck patients in seated position and report larger position variations of up to several millimeters in some cases. In order to restrain patient mobility within the masks, additional immobilization devices may hence be needed. It has to be noted that the design by Balakin et al. considered a straight upright posture, which was found sub-optimal regarding patient motion in (31).

Buchner et al. (35) present the development of soft robots as immobilization devices to counter patient slouching in the seated position. The soft robots based on fluid-driven origami inspired artificial muscles have the advantage of being highly adaptive, providing individually optimized support for the patient. Hence, the soft robot technology may present several advantages over conventional immobilization devices for upright positioning. For example, they can provide the best position for the day, without the need to make and store several immobilization devices. Buchner et al. report promising results for immobilization and positioning with their device.

To fixate the pelvis and thereby stabilise the upper body, Leo Cancer Care Ltd. (8) have proposed a knee rest and heel fixation, using the patient’s femur to push the pelvis into the chair seat. Such a device might provide stability for avoiding patient sagging, removing load from upper body or head fixation masks. It can be speculated that the knee support may also benefit the reproducibility of the femur-hip angle, which is a known issue for particle prostate therapy (53).

Mackie *et al.* (21) used a prototype of the Leo Cancer Care upright positioning system to investigate positioning accuracy for different treatment sites with an optical system. Deviations in setup accuracy for thorax patients were comparable to those found in literature for supine treatments. It should be noted that they measured external motion shifts only, but not potential motion of the tumor inside the body, which might differ from supine positions.

Most recently, Sun et al. (31) published a clinical study on the first experience for patient intra- and inter-treatment position changes with the SPHIC chair system, reporting an accuracy comparable to that of traditional supine positioning. Similar, McCarroll et al. (26) for their forward leaning design report head&neck positioning accuracy comparable to that in traditional supine position, except for one patient, where large displacement in the order of centimeters was observed.

Similar to a conventional couch system, any upright positioning device requires regular quality assurance to verify its required clinical accuracy better than 0.5°/1 mm (54). Especially for 6DOF support devices consisting of multiple individual components, agreement of their intrinsic coordinate systems with each other must be ensured, and potential variations be accurately corrected for, in order to avoid error propagation (55). reported a framework for coordinate system alignment and correction of systematic errors in the movement of the SPHIC treatment chair. Quality assurance reported for chair systems, so far, considered rigid phantoms (e.g., in (46)). However, to ensure quality of immobilization devices for upright positioning, one may also need to consider the support against deformations in upright posture. New, targeted quality assurance strategies may therefore be advisory.

## Image Guidance for Upright Positions

Significant anatomical variation can be expected for nearly all treatment sites, with the possible exception of the brain as it is enclosed in the rigid skull. The relative change of the gravity vector and the accompanying change in muscle tension will cause non-rigid changes to most organs. Yan et al. (56) investigated patient alignment between supine and upright (slightly reclined) position using an upright MRI scanner. They report good alignment for the head, but already note deformations in the neck region. Organs in abdomen or thorax are associated with larger differences between supine and upright (28, 56, 57). The success of upright treatment therefore crucially depends on the availability of an upright CT scanner for treatment simulation.

A dedicated x-ray CT system for upright treatments was implemented by Kamada et al. (5). The system was attached to the ceiling of the treatment room, and lowered down over the positioned patient. A contact sensor on the lower surface of the scanner was used to avoid collisions with the chair or patient. Shah et al. (58) report the use of a vertical CT scanner at Fermi Lab for a special patient case that could not tolerate supine positioning due to their medical condition. Schreuder (30) designed a system for vertical CT imaging from any angle between vertical and horizontal. P-Cure Ltd (9) offers a commercial system for upright CT imaging, which was installed at the Northwestern Medicine Chicago Proton Center in 2016, and has been used in clinical routine since then. However, to the best of the authors knowledge, no study on the system performance, nor patient data is yet presented in literature. Notably, for sitting patient postures, i.e., those with legs in ~90° angle to the torso, imaging below the diaphragm is not possible due to the limited CT bore diameter. Most recently Leo Cancer Care Ltd (8) have proposed a design for a rotatable Dual-Energy CT scanner. The system, which is a follow up of the design presented by Schreuder (30), is intended to be mounted between two supporting columns with a central rotation axis, enabling imaging at any angle between supine to upright position. As for previous vertical CT designs, the system would then slide over the patient for image acquisition. It is intended to enable full CT imaging of treatment sites down to the pelvis in combination with a half standing or standing patient posture as provided by their upright positioning device.

While a vertical x-ray CT scanner positioned directly at isocenter may provide the greatest accuracy, it may not be feasible due to space limitations, especially above the isocenter. In addition, if a switch to a treatment couch is desired, installation of a vertical x-ray CT on columns lateral to the isocenter may be challenging due to the lateral clearance (±2m) needed for full couch motion. If setup uncertainties between an out-of-room planning vertical CT are deemed too large, or no space for a second upright positioning system with vertical CT is available, in-room x-ray CT not at isocenter may be more practical. In a recent study, Nesteruk et al. (59) have found no significant difference between in-isocenter cone-beam CT (CBCT) based treatment planning, and treatment planning based on a CT-on-rails for supine position. The same may be the case for upright, such that an in-room CT instead of one at isonceter may provide sufficient accuracy. This then requires a suitable option to move the chair between CT and isocenter is available (robot arm, rail).

CBCT at isocenter also presents a viable option for verification, as done in (31), and plan simulation. The flexibility to rotate the patient instead of the imaging device for CBCT acquisition has already been explored in (60) and was recently taken into account for the design of the MARIE™ proton therapy solution by Leo Cancer Care Ltd (8). If options for upright and supine positioning are foreseen, robotic CBCT systems should be able to provide the necessary flexibility, especially since they allow for non-co-planar image acquisition (61). CBCT may be a more practical solution for imaging at isocenter compared to vertical CT, not least with the recent boost in achievable CBCT image accuracy *via* iterative and machine learning based algorithms (7). Even accurate 4D-CBCT based proton therapy dose calculations were made possible (62), making CBCT particularly attractive for upright particle therapy.

Treatment planning typically involves modalities beyond X-ray CT, especially for contouring of target volumes and organs at risk, such as MRI or PET. A vertical PET for the patient in seated position was already considered by Heeg et al. (6). Upright MRI systems have been used in various studies for investigating anatomical differences between upright and supine positions (28, 29, 56, 57, 63). To provide best possible accuracy, having both the MRI and x-ray CT in upright posture would be preferential, as increased uncertainties in the deformable registration between the two postures are to be expected. Still, vertical MRI and PET scanners with the same image quality as current clinical systems for recumbent patients might be difficult to achieve, due to the size and geometry of the involved detector and acquisition systems. The viability of upright treatments considering the whole treatment planning chain therefore needs to be carefully investigated (23).

Particle radiography (pRad) and particle CT (pCT) present interesting options for at-isocenter imaging in particle therapy, and are particularly suited for upright patient positions. For particle imaging, the particle beam energy is increased beyond the therapeutic level such that the particles fully cross the patient, which enables to reconstruct images of the patient’s integral water equivalent thickness (64, 65). pRad has shown good capability for position (66, 67) and anatomy (68) verification from beams eye view, and for optimization of the x-ray CT relative stopping power calibration (69–71). By rotating the patient, full pCT scans may be acquired. pCT scans may be used directly for treatment planning, where the direct nature of assessing the relative stopping power provides high stopping power and range prediction accuracy (72–75). pCT and upright treatment posture are inherently a well suited match, since pCT acquisition would be easier with a fixed beam and detector setup and rotating patient compared to acquisition with a rotating gantry. In fact, all currently existing pCT prototypes use rotating platforms to rotate the object for pCT acquisition. Moreover, the absence of beam hardening or metal artifacts for pCT (76) could prove useful for upright treatments, as it permits more flexible choice of materials for the immobilization devices and still obtain artifact free images.

Optical guidance systems are suitable to quickly verify the patient position. Commercial optical guidance systems have already been the system of choice to verify the accuracy of upright positioning solutions for different studies found in literature (21, 35, 45, 46) As such, an optical tracking system may be considered as a standard with upright treatment postures.

In terms of treatment plan simulation and optimization, the same infrastructure can be used for both upright and supine/prone positions, through the introduction of a simple coordinate transformation, as introduced by Krämer et al. (77) to the experimental treatment planning platform TRiP98 (78). This is advantageous regarding the introduction of an upright positioning system into daily clinical routine. Recently, an option for a seated patient position was implemented in the commercially available treatment planning system RayStation (RaySearch Laboratories, Stockholm, Sweden) for proton therapy and was validated with a commercial radiotherapy chair solution offered by Q-fix (Avondale, Pennsylvania, USA) (79). Hegarty et al. (17) note additional constraints concerning the patient tolerance of rotational speed and acceleration of the chair during treatment planning for photon IMRT or VMAT treatments. This is of less relevance for particle therapy, which is typically applied through few fields between which the chair rotation can be comfortably adapted. Nevertheless, this would need to be considered in the context of particle arc therapy, where varying rotational speed during delivery is present (16).

## Discussion

In the following, we briefly summarize the key points to take from previous literature. Then we present some recommendations for future systems, where we distinguish between retrofitting an upright positioning system into an existing facility and designing a new facility upfront with upright positioning.

### Key Take-Aways From Literature

It is evident from literature, that a chair system aimed for different treatment sites needs to be highly modular and flexible. Different postures, ranges of motion and fixation devices are required for different treatment sites, with the optimal solution for each site still being subject of further research. While a one-fits-all upright patient positioning system would certainly be most efficient in terms of clinical workflow, it might not necessarily be the best solution for each site. For example, a mounting mechanism for the patient mask made of steel provides excellent accuracy for head&neck treatment (39, 46). But steel in the backrest would likely prohibit thoracic treatments, due to the CT artifacts and risk of activation that this entails. Moreover, an upright treatment will not be suited for all patients, who would then be excluded by a chair-only solution (26).

The most important question to ask prior to designing a chair system is therefore which patient cohorts are to be treated. A clinic aiming to be the main particle therapy provider for a large geographical region will have different aspirations compared to a specialized treatment facility in a region with existing alternative centers. A similar argument can also be made regarding the number of available treatment rooms in the facility.

A key challenge for upright treatments that has been pointed out in several publications is the different requirements placed on immobilization devices. While an inaccurate position, for example from mechanical sagging of the chair, may be corrected through adequate quality assurance (45), individual sagging of the patients in the immobilization devices would necessitate online image guidance to be detected, as well as a framework for real-time adaptive radiotherapy to be corrected for. Immobilization devices therefore should be a center point in the design of new upright positioning systems.

In the same line of thought, vertical imaging is paramount to the success of upright treatment. While conventional x-ray radiography image guidance systems have been used successfully in (31) for patient alignment, CBCT would be better and more flexible. In-room CT or in-isocenter CT may not be necessary for patient positioning, however, vertical, planning-quality CT imaging needs to be available somewhere in the facility, if patient sites other than the head are to be treated. Optical surface guidance, in addition, has shown to be a useful tool for verifying and monitoring upright patient position.

Particle CT could be a well suited candidate for in-isocenter CT with limited available space. However, long scan acquisition times and the need for higher-than-treatment beam energies are still limiting factors (80). With advances in technology, particle CT could be the preferred option of choice compared to in-isocenter x-ray CT.

In terms of posture, there is currently too little evidence to conclude on an ideal posture for the different indications beside head&neck, for which a slightly reclined posture seems to be ideal. For treatments at or above the thorax, any position that enables secure fixation of the pelvis in a reproducible position may work to restrain patient sagging. For treatment below diaphragm, however, a half-standing or standing posture is the only feasible option.

At least initially, the limited experience on anatomical changes for upright treatments will likely necessitate the use of increased safety margins. These, however, will be reduced with increasing experience with the upright position. Frequent, and ideally, isocentric planning-quality image guidance should be performed during the pioneering phase of upright treatments.

### Retrofitting an Upright Positioning System to an Existing Facility

When retrofitting an upright positioning system to an existing (fixed beam line) facility, the major limiting factor is the available space. Equipment, like a couch robot, already in the treatment room needs to be considered and the fixed floor-isocenter-ceiling heights pose a major constraint.

For a multi-room facility, a dedicated specialized treatment room with an upright positioning system, where existing equipment for recumbent positioning may be discarded, could be a viable option. For single- or two-room facilities, the capability to switch to a supine/prone treatment position is a key to ensure efficacy. The most preferential/cost-efficient solution would hence be one that can be coupled to the existing robot positioning devices used already for the treatment couch. The robot arm could be used to also mount or park the individual modules for easier handling (30).

Having both a couch and upright treatment positioning system in isocenter makes the additional installation of an in-isocenter vertical CT, capable of imaging in both postures, challenging, due to the clearance required for couch motion. If not enough room for an in-isocenter, or in-room rotatable CT is available, at least x-ray radiography image guidance or CBCT suitable for upright position verification appears mandatory. Due to the flexible acquisition trajectories, a C-arm CBCT system suitable for both couch and upright positioning system would be the preferred option.

For accurate simulation, a vertical CT will be necessary for most treatment sites. Even if an in-room vertical CT exists, a more efficient workflow might be achievable if space for an upright CT scanner and upright positioning system was available somewhere in the facility for simulation. Otherwise, the treatment room would be blocked for planning CT acquisition. These issues should be thoroughly considered with the clinical staff before installation of an upright patient positioning system. Due to the missing constraint placed by the fixed beam height, a not-in-isocenter vertical CT requires lower ceiling height compared to one positioned at isocenter, which renders installation easier.

### Designing a New Facility With an Upright Positioning System

Modeling of patient numbers, treatments sites and division of patients to the different rooms should be carried out carefully. The chair design greatly depends on the intended treatment sites, which also dictates different options for imaging and necessary range of motion. A restriction to certain treatments can greatly reduce cost, not only in the chair itself, but especially in terms of room size. However, a too narrow specification might hinder an efficient workflow later on, and prove even more costly long term.

Again, a central point is the number of rooms to be available at the facility. If multiple rooms are planned, a dedicated room for upright treatments could be designed. Here, the choice of 6DOF support structure would not need to consider space limitations nor a switch to couch. This room also should feature enough space to accommodate in-isocenter image guidance systems. The facility design should also consider optimizing the height of the isocenter with respect to the upright positioning system. This should maximize the number of patients that can be treated for the intended body sites, while minimizing the displacement required of the chair positioning system, especially for hexapods with limited workspace.

All current single room designs feature a gantry. A fixed beam single room with a chair would likely be possible on a previously not achievable low budget, so that also a highly specialized solution may become plausible in the future. This could focus only on a select subset of patients, such as only thoracic and H&N patients, and could offer a dedicated solution for this sub-group. As such, it could potentially offer treatment quality for its patients comparable to a gantry-based system, but at lower cost. However, careful modeling would be necessary to judge clinical and commercial viability.

Additional space for a vertical CT should be planned, or patients would have to be positioned without planning quality imaging. This might be challenging especially to early adopters, when clinical experience is still limited. A separate vertical CT scanner that can enable imaging both in upright and supine position would ensure highest efficacy. Installation of an open bore/upright MRI at the facility could be considered, but further studies on errors in deformable registration between recumbent and upright positions are needed to support any decision.

## Conclusion

Upright patient positioning has distinct economical and clinical benefits that may make it a key technology for the next generation particle therapy facilities. Still, an upright positioning system brings many clinical and also engineering challenges, to achieve highly accurate and stable patient positioning. In addition, limited experience in the difference between patient anatomy in supine or upright position is available. However, we are currently seeing a boost in knowledge with several developments towards upright patient positioning systems driven by the need to reduce particle therapy cost and increase efficiency. In this review, we aimed at highlighting key points from these developments to make recommendations for an ideal upright system. It seems that a one-fits-all solution is hard or even impossible to achieve. The clinical benefit of an upright positioning device therefore needs to be thoroughly evaluated for different patient sites to identify, where the upright position would provide the greatest benefit, and to tailor the patient positioning system design. Such studies are currently underway, and we are expecting exciting developments of upright positioning systems that will bring a paradigm shift for the future of particle therapy.

## Author Contributions

LV — conceptualization, literature review, writing (original draft), writing (revision). YS — conceptualization, writing (revision). MD — conceptualization, supervision, funding acquisition, writing (revision). CG — conceptualization, supervision, funding acquisition, writing (original draft), writing (revision). All authors contributed to the article and approved the submitted version.

## Funding

This project has received funding from the European Union’s Horizon 2020 research and innovation programme under grant agreement No 101008548.

## Conflict of Interest

Authors LV, YS, MD and CG were employed by GSI Helmholtz Center for Heavy Ion Research GmbH.

## Publisher’s Note

All claims expressed in this article are solely those of the authors and do not necessarily represent those of their affiliated organizations, or those of the publisher, the editors and the reviewers. Any product that may be evaluated in this article, or claim that may be made by its manufacturer, is not guaranteed or endorsed by the publisher.

## References

[1] Particle Therapy Co-Operative Group. Particle Therapy Facilities in Clinical Operation (Last Update: (November 2021) (2021). Available at: https://www.ptcog.ch/index.php/patient-statistics.

[2] Durante M Paganetti H. Nuclear Physics in Particle Therapy: A Review. Rep Prog Phys (2016) 79:96702. doi: 10.1088/0034-4885/79/9/096702 27540827

[3] Paganetti H Beltran C Both S Dong L Flanz J Furutani K. Roadmap: Proton Therapy Physics and Biology. Phys Med Biol (2021) 66(5):05RM01. doi: 10.1088/1361-6560/abcd16. PMC9275016 33227715

[4] Jäkel O Kraft G Karger CP. The History of Ion Beam Therapy in Germany. Z für Med Physik (2022) 32:6–22. doi: 10.1016/j.zemedi.2021.11.003 PMC9948864 35101337

[5] Kamada T Tsujii H Mizoe JE Matsuoka Y Tsuji H Osaka Y. A Horizontal Ct System Dedicated to Heavy-Ion Beam Treatment. Radiother Oncol (1999) 50:235–7. doi: 10.1016/S0167-8140(99)00005-5 10368048

[6] Heeg P Kuhn S Schardt D Schultz-Ertner D. A Treatment Chair for the Therapy Facility. In: GSI Report, vol. vol. 2000-1. . Darmstadt: GSI (2000). p. 167. Wissenschaftlicher Ergebnisbericht der GSI, GSI Annual Report.

[7] Landry G Hua Ch.. Current State and Future Applications of Radiological Image Guidance for Particle Therapy. Med Phys (2018) 45:e1086–95. doi: 10.1002/mp.12744 30421805

[8] Leo Cancer Care Ltd. Leo Cancer Care Ltd. Website (2021). Available at: www.leocancercare.com (Accessed Dec. 9, 2021).

[9] P-cure. Patient Centric Proton Therapy Website (2021). Available at: www.p-cure.com (Accessed Dec. 9, 2021).

[10] Bortfeld TR MFd V Yan S. The Societal Impact of Ion Beam Therapy. Z fur med Physik (2021), 31(2):102–104. doi: 10.1016/j.zemedi.2020.06.007 PMC7361098 32680688

[11] Mazal A Vera Sanchez JA Sanchez-Parcerisa D Udias JM España S Sanchez-Tembleque V. Biological and Mechanical Synergies to Deal With Proton Therapy Pitfalls: Minibeams, Flash, Arcs, and Gantryless Rooms. Front Oncol (2021) 10:613669. doi: 10.3389/fonc.2020.613669 33585238 PMC7874206

[12] Durante M Debus J Loeffler JS. Physics and Biomedical Challenges of Cancer Therapy With Accelerated Heavy Ions. Nat Rev Phys (2021) 3:777–90. doi: 10.1038/s42254-021-00368-5 PMC7612063 34870097

[13] Parodi K Polf JC. *In Vivo* Range Verification in Particle Therapy. Med Phys (2018) 45:e1036–50. doi: 10.1002/mp.12960 PMC6262833 30421803

[14] Lis M Newhauser W Donetti M Wolf M Steinsberger T Paz A. Dosimetric Validation of a System to Treat Moving Tumors Using Scanned Ion Beams That are Synchronized With Anatomical Motion. Front Oncol (2021) 11:712126. doi: 10.3389/fonc.2021.712126 34568041 PMC8456027

[15] Paganetti H Botas P Sharp GC Winey B. Adaptive Proton Therapy. Phys Med Biol (2021) 66:22TR01. doi: 10.1088/1361-6560/ac344f PMC8628198 34710858

[16] Li X Liu G Janssens G De Wilde O Bossier V Lerot X. The First Prototype of Spot-Scanning Proton Arc Treatment Delivery. Radiother Oncol (2019) 137:130–6. doi: 10.1016/j.radonc.2019.04.032 31100606

[17] Hegarty S Hardcastle N Korte J Kron T Everitt S Rahim S. Please Place Your Seat in the Full Upright Position: A Technical Framework for Landing Upright Radiation Therapy in the 21st Century. Front Oncol (2022) 12:821887. doi: 10.3389/fonc.2022.821887 35311128 PMC8929673

[18] Moyers MF Lesyna W. Isocenter Characteristics of an External Ring Proton Gantry. Int J Radiat Oncol Biol Phys (2004) 60:1622–30. doi: 10.1016/j.ijrobp.2004.08.052 15590194

[19] Haberer T Debus J Eickhoff H Jäkel O Schulz-Ertner D Weber U. The Heidelberg Ion Therapy Center. Radiother Oncol (2004) 73:S186–90. doi: 10.1016/S0167-8140(04)80046-X 15971340

[20] Bottura L Felcini E De Rijk G Dutoit B. Gatoroid: A Novel Toroidal Gantry for Hadron Therapy. Nucl Instrum Methods Phys Res Section A: Accelerators Spectrometers Detectors Associated Equip (2020) 983:164588. doi: 10.1016/j.nima.2020.164588

[21] Mackie TR Towe S Schreuder N. Is Upright Radiotherapy Medically and Financially Better? AIP Conf Proc (2021) 2348:020002. doi: 10.1063/5.0051770

[22] Yan S Lu HM Flanz J Adams J Trofimov A Bortfeld T. Reassessment of the Necessity of the Proton Gantry: Analysis of Beam Orientations From 4332 Treatments at the Massachusetts General Hospital Proton Center Over the Past 10 Years. Int J Radiat Oncol Biol Phys (2016) 95:224–33. doi: 10.1016/j.ijrobp.2015.09.033 26611874

[23] Schreuder N Ding X Li Z. Fixed Beamlines can Replace Gantries for Particle Therapy. Med Phys (2022) 49(4):2097–2100. doi: 10.1002/mp.15531 35147222

[24] Yan S Depauw N Adams J Gorissen BL Shih HA Flanz J. Technical Note: Does the Greater Power of Pencil Beam Scanning Reduce the Need for a Proton Gantry? study head-and-neck Brain tumors Med Phys (2022) 49:813–24. doi: 10.1002/mp.15409 34919736

[25] Court L Yang J Fullen D Han N Ko J Mason S. Su-E-T-359: Patients Could (and Should) be Treated in an Upright Position. Med Phys (2013) 40:287–7. doi: 10.1118/1.4814793

[26] McCarroll RE Beadle BM Fullen D Balter PA Followill DS Stingo FC. Reproducibility of Patient Setup in the Seated Treatment Position: A Novel Treatment Chair Design. J Appl Clin Med Phys (2017) 18:223–9. doi: 10.1002/acm2.12024 PMC5689874 28291911

[27] Dellamonica J Lerolle N Sargentini C Hubert S Beduneau G Marco FD. Effect of Different Seated Positions on Lung Volume and Oxygenation in Acute Respiratory Distress Syndrome. Intensive Care Med (2013) 39:1121–7. doi: 10.1007/s00134-013-2827-x 23344832

[28] Yang J Chu D Dong L Court LE. Advantages of Simulating Thoracic Cancer Patients in an Upright Position. Pract Radiat Oncol (2014) 4:e53–8. doi: 10.1016/j.prro.2013.04.005 24621432

[29] von Siebenthal M Székely G Lomax AJ Cattin PC. Systematic Errors in Respiratory Gating Due to Intrafraction Deformations of the Liver. Med Phys (2007) 34:3620–9. doi: 10.1118/1.2767053 17926966

[30] Schreuder AN. Technological Developments Allowing For The Widespread Clinical Adoption Of Proton Radiotherapy. London, United Kingdom: University College London (2020).

[31] Sun J Kong L Chen Z You D Mao J Guan X. Clinical Implementation of a 6d Treatment Chair for Fixed Ion Beam Lines. Front Oncol (2021) 11:694749. doi: 10.3389/fonc.2021.694749 34249751 PMC8260974

[32] Hrbacek J Mishra KK Kacperek A Dendale R Nauraye C Auger M. Practice Patterns Analysis of Ocular Proton Therapy Centers: The International Optic Survey. Int J Radiat Oncol Biol Phys (2016) 95:336–43. doi: 10.1016/j.ijrobp.2016.01.040 27084651

[33] Ciocca M Magro G Mastella E Mairani A Mirandola A Molinelli S. Design and Commissioning of the non-Dedicated Scanning Proton Beamline for Ocular Treatment at the Synchrotron-Based Cnao Facility. Med Phys (2019) 46:1852–62. doi: 10.1002/mp.13389 30659616

[34] Rahim S Korte J Hardcastle N Hegarty S Kron T Everitt S. Upright Radiation Therapy—a Historical Reflection and Opportunities for Future Applications. Front Oncol (2020) 10:213. doi: 10.3389/fonc.2020.00213 32158693 PMC7052284

[35] Buchner T Yan S Li S Flanz J Hueso-González F Kielty E. (2020). A Soft Robotic Device for Patient Immobilization in Sitting and Reclined Positions for a Compact Proton Therapy System, in: 8th IEEE RAS/EMBS International Conference for Biomedical Robotics and Biomechatronics (BioRob), pp. 981–8. doi: 10.1109/BioRob49111.2020.9224389

[36] Castro JR Quivey JM Lyman JT Chen GTY Tobias CA Kanstein LL. Heavy Ion Therapy (Division of Biological and Environmental Research U.S Vol. vol. LBL-5610. Energy Research and Development Administration (1977) p. 198–218.

[37] Castro JR Quivey JM Lyman JT Chen GTY Phillips TL Tobias CA. Current Status of Clinical Particle Radiotherapy at Lawrence Berkeley Laboratory. Cancer (1980) 46:633–41. doi: 10.1002/1097-0142(19800815)46:4<633::AID-CNCR2820460402>3.0.CO;2-O 6772294

[38] Gunn JT Lyman JT. A Versatile Patient Positioner for Radiation Therapy. IEEE Trans Nucl Sci (1973) 20:1022–4. doi: 10.1109/TNS.1973.4327314

[39] Heeg P Schardt D Störmer J. The Patient Fixation Unit for the Treatment Chair in Cave M. In: GSI Report, vol. vol. 2002-1. . Darmstadt: GSI (2002). p. 279 p.

[40] Lis M Newhauser W Donetti M Durante M Weber U Zipfel B. A Facility for the Research, Development, and Translation of Advanced Technologies for Ion-Beam Therapies. J Instrum (2021) 16:T03004. doi: 10.1088/1748-0221/16/03/t03004

[41] Marash M Shpunt M. . Apparatus and method for providing patient imaging (2013). U.S. patent application US20150208992A1; Assignee: P-Cure Ltd.

[42] Balakin VE Belikhin MA Pryanichnikov AA Shemyakov AE Strelnikova NS. Clinical Application of New Immobilization System in Seated Position for Proton Therapy. KnE Energy (2018) 3:45–51. doi: 10.18502/ken.v3i2.1790

[43] Balakin V. Charged Particle Treatment, Rapid Patient Positioning Apparatus and Method of Use Thereof (2012). , US patent US9056199B2; Assignee: V. A. Balakin, V. P. Balakin.

[44] Zhang Y Yang Z Jiang J Dai X Qin P Guo S. Design Analysis and Experimental Study of Robotic Chair for Proton Heavy Ion Radiotherapy. Appl Bionics Biomech (2019) 2019:6410941. doi: 10.1155/2019/6410941 31885688 PMC6914987

[45] Zhang X Hsi WC Yang F Wang Z Sheng Y Sun J. Development of an Isocentric Rotating Chair Positioner to Treat Patients of Head and Neck Cancer at Upright Seated Position With Multiple Nonplanar Fields in a Fixed Carbon-Ion Beamline. Med Phys (2020) 47:2450–60. doi: 10.1002/mp.14115 32141079

[46] Sheng Y Sun J Wang W Stuart B Kong L Gao J. Performance of a 6d Treatment Chair for Patient Positioning in an Upright Posture for Fixed Ion Beam Lines. Front Oncol (2020) 10:122. doi: 10.3389/fonc.2020.00122 32117769 PMC7026365

[47] BEC GmbH. Exacure Website (2021). Available at: www.exacure.com (Accessed Dec. 21, 2021).

[48] Bouraoui MW Buck M. Sitzvorrichtung Für Eine Patientenpositioniereinrichtung Und Patientenpositioniereinrichtung Mit Einer Sitzvorrichtung. BEC GmbH (2018). Available at: https://depatisnet.dpma.de/DepatisNet/depatisnet?action=bibdat&docid=DE102016015530A1

[49] Jürgens H Matzdorff I Windberg J. International Anthropometric Data for Work-Place and Machinery Design. Arbeitswissenschaftliche Erkenntnisse, Dortmund, Germany: Bundesanstalt für Arbeitsschutz und Arbeitsmedizin Vol. 108. (1998). p. 11. Available at: https://www.baua.de/DE/Angebote/Publikationen/AWE/AWE108e.html

[50] Feain I Towe S Strangeman M. Patient Positioning Apparatus. Leo Cancer Care Ltd (2017).

[51] Comission IE. Medical Electrical Equipment – ALL PARTS. Geneva, CH: International standard, International Electronical Commission (2021).

[52] Nairz O Winter M Heeg P Jäkel O. Accuracy of Robotic Patient Positioners Used in Ion Beam Therapy. Radiat Oncol (2013) 8:124. doi: 10.1186/1748-717X-8-124 23692666 PMC3749753

[53] Trofimov A Nguyen PL Efstathiou JA Wang Y Lu HM Engelsman M. Interfractional Variations in the Setup of Pelvic Bony Anatomy and Soft Tissue, and Their Implications on the Delivery of Proton Therapy for Localized Prostate Cancer. Int J Radiat oncol biol Phys (2011) 80:928–37. doi: 10.1016/j.ijrobp.2010.08.006 PMC3026870 20947266

[54] Klein EE Hanley J Bayouth J Yin FF Simon W Dresser S. Task Group 142 Report: Quality Assurance of Medical Acceleratorsa). Med Phys (2009) 36:4197–212. doi: 10.1118/1.3190392 19810494

[55] Zhang X Hsi W Yang F Zhou R. Verification and Validation (VnV) of Control Software for an Isocentric Rotation Chair Positioner Used to Treat Patients With Head/Neck Cancers in a Fixed Carbon-Ion Beamline. J Instrum (2022) 17:P01021. doi: 10.1088/1748-0221/17/01/p01021

[56] Yan S Greenhalgh J Li S Bortfeld T Flanz J. (2020). An Mri Study of Organ Shape Variations Between Upright, Reclined and Recumbent Positions: Implications for Compact Gantry-Less Particle Therapy, in: Proceedings to the 58th Annual Conference of the Particle Therapy Cooperative Group (PTCOG58), International Journal of Particle Therapy Vol. 6. pp. 45–491. doi: 10.14338/IJPT.19-PTCOG-6.4

[57] Hayes AR Gayzik FS Moreno DP Martin RS Stitzel JD. Comparison of Organ Location, Morphology, and Rib Coverage of a Midsized Male in the Supine and Seated Positions. Comput Math Methods Med (2013) 2013:419821. doi: 10.1155/2013/419821 23606901 PMC3623390

[58] Shah AP Strauss JB Kirk MC Chen SS Kroc TK Zusag TW. Upright 3d Treatment Planning Using a Vertical Ct. Med Dosimetry (2009) 34:82–6. doi: 10.1016/j.meddos.2008.05.004 19181260

[59] Nesteruk KP Bobić M Lalonde A Winey BA Lomax AJ Paganetti H. Ct-On-Rails Versus in-Room Cbct for Online Daily Adaptive Proton Therapy of Head-and-Neck Cancers. Cancers (2021) 13(23). doi: 10.3390/cancers13235991 PMC8656713 34885100

[60] Fave X Yang J Carvalho L Martin R Pan T Balter P. Upright Cone Beam Ct Imaging Using the Onboard Imager. Med Phys (2014) 41:061906. doi: 10.1118/1.4875682 24877817

[61] Hatamikia S Biguri A Kronreif G Kettenbach J Russ T Furtado H. Optimization for Customized Trajectories in Cone Beam Computed Tomography. Med Phys (2020) 47:4786–99. doi: 10.1002/mp.14403 PMC7693244 32679623

[62] Schmitz H Rabe M Janssens G Bondesson D Rit S Parodi K. Validation of Proton Dose Calculation on Scatter Corrected 4d Cone Beam Computed Tomography Using a Porcine Lung Phantom. Phys Med Biol (2021) 66:175022. doi: 10.1088/1361-6560/ac16e9 34293737

[63] Abdulaziz M Kavanagh A Stothers L Macnab AJ. (2018). Relevance of Open Magnetic Resonance Imaging Position (Sitting and Standing) to Quantify Pelvic Organ Prolapse in Women, in: Canadian Urological Association journal = Journal de l’Association des urologues du Canada, , Vol. 12. pp. E453–60. doi: 10.5489/cuaj.5186 PMC6217953 29989885

[64] Collins-Fekete CA Brousmiche S Portillo SKN Beaulieu L Seco J. A Maximum Likelihood Method for High Resolution Proton Radiography/Proton CT. Phys Med Biol (2016) 61:8232. doi: 10.1088/0031-9155/61/23/8232 27811399

[65] Deffet S Farace P Macq B. Sparse Deconvolution of Proton Radiography Data to Estimate Water Equivalent Thickness Maps. Med Phys (2020) 47:509–17. doi: 10.1002/mp.13917 31705805

[66] Palaniappan P Meyer S Kamp F Belka C Riboldi M Parodi K. Deformable image registration of the treatment planning CT with proton radiographies in perspective of adaptive proton therapy. Physics in Medicine & mathsemicolon Biology (2021) IOP Publishing 66:045008. doi: 10.1088/1361-6560/ab8fc3 32365335

[67] Deffet S Macq B Righetto R Vander Stappen F Farace P. Registration of Pencil Beam Proton Radiography Data With X-Ray Ct. Med Phys (2017) 44:5393–401. doi: 10.1002/mp.12497 28771749

[68] Sarosiek C DeJongh EA Coutrakon G DeJongh DF Duffin KL Karonis NT. Analysis of Characteristics of Images Acquired With a Prototype Clinical Proton Radiography System. Med Phys (2021) 48:2271–8. doi: 10.1002/mp.14801 PMC8141022 33621368

[69] Collins-Fekete CA Brousmiche S Hansen DC Beaulieu L Seco J. Pre-Treatment Patient-Specific Stopping Power by Combining List-Mode Proton Radiography and X-Ray Ct. Phys Med Biol (2017) 62:6836. doi: 10.1088/1361-6560/aa7c42 28657550

[70] Gianoli C Göppel M Meyer S Palaniappan P Rädler M Kamp F. Patient-Specific Ct Calibration Based on Ion Radiography for Different Detector Configurations in 1h, 4he and 12c Ion Pencil Beam Scanning. Phys Med Biol (2020) 65(24):245014. doi: 10.1088/1361-6560/aba319 32629442

[71] Meijers A Free J Wagenaar D Deffet S Knopf AC Langendijk JA. Validation of the Proton Range Accuracy and Optimization of CT Calibration Curves Utilizing Range Probing. Phys Med Biol (2020) 65:03NT02. doi: 10.1088/1361-6560/ab66e1 31896099

[72] Meyer S Kamp F Tessonnier T Mairani A Belka C Carlson DJ. Dosimetric Accuracy and Radiobiological Implications of Ion Computed Tomography for Proton Therapy Treatment Planning. Phys Med Biol (2019) 64:125008. doi: 10.1088/1361-6560/ab0fdf 30870831

[73] Dedes G Dickmann J Niepel K Wesp P Johnson RP Pankuch M. Experimental Comparison of Proton CT and Dual Energy X-Ray CT for Relative Stopping Power Estimation in Proton Therapy. Phys Med Biol (2019) 64:165002. doi: 10.1088/1361-6560/ab2b72 31220814

[74] Bär E Volz L Collins-Fekete CA Brons S Runz A Schulte RW. Experimental Comparison of Photon Versus Particle Computed Tomography to Predict Tissue Relative Stopping Powers. Med Phys (2022) 49:474–87. doi: 10.1002/mp.15283 PMC13655494 34709667

[75] Volz L Collins-Fekete CA Bär E Brons S Graeff C Johnson RP. The Accuracy of Helium Ion CT Based Particle Therapy Range Prediction: An Experimental Study Comparing Different Particle and X-Ray CT Modalities. Phys Med Biol (2021) 66:235010. doi: 10.1088/1361-6560/ac33ec PMC8792995 34706355

[76] Civinini C Scaringella M Brianzi M Intravaia M Randazzo N Sipala V. Relative Stopping Power Measurements and Prosthesis Artifacts Reduction in Proton {CT}. Phys Med Biol (2020) 65:225012. doi: 10.1088/1361-6560/abb0c8 33200747

[77] Krämer M Grözinger SO Hasch B Jäkel O Heeg P. Treatment Planning for the GSI Radiotherapy. In: GSI Report, vol. vol. 2005-1. . Darmstadt: GSI (2005). p. 496 p.

[78] Krämer M Jkel O Haberer T Kraft G Schardt D Weber U. Treatment Planning for Heavy-Ion Radiotherapy: Physical Beam Model and Dose Optimization. Phys Med Biol (2000) 45:3299–317. doi: 10.1088/0031-9155/45/11/313 11098905

[79] Maes D Janson M Regmi R Egan A Rosenfeld A Bloch C. Validation and Practical Implementation of Seated Position Radiotherapy in a Commercial Tps for Proton Therapy. Phys Med: Eur J Med Phys (2020) 80:175–85. doi: 10.1016/j.ejmp.2020.10.027 33189048

[80] Volz L Collins-Fekete CA Piersimoni P Seco J. Recent Developments in Proton Imaging. CRC Press, Boca Raton (2021) p. 64–82. chap. 5. doi: 10.1201/9781003212485

